# Skin Disease in Children: Effects on Quality of Life, Stigmatization, Bullying, and Suicide Risk in Pediatric Acne, Atopic Dermatitis, and Psoriasis Patients

**DOI:** 10.3390/children8111057

**Published:** 2021-11-16

**Authors:** Katherine A. Kelly, Esther A. Balogh, Sebastian G. Kaplan, Steven R. Feldman

**Affiliations:** 1 Center for Dermatology Research, Department of Dermatology, Wake Forest School of Medicine, Winston-Salem, NC 27103, USA; ebalogh@wakehealth.edu (E.A.B.); sfeldman@wakehealth.edu (S.R.F.); 2 Department of Psychiatry, Wake Forest School of Medicine, Winston-Salem, NC 27103, USA; sgkaplan@wakehealth.edu; 3 Department of Social Sciences & Health Policy, Wake Forest School of Medicine, Winston-Salem, NC 27103, USA; 4 Department of Dermatology, University of Southern Denmark, 5230 Odense, Denmark; 5 Department of Pathology, Wake Forest School of Medicine, Winston-Salem, NC 27103, USA

**Keywords:** self-esteem, acne, atopic dermatitis, psoriasis, pediatrics, stigmatization, suicide, quality of life

## Abstract

Acne, atopic dermatitis (AD), and psoriasis are all chronic dermatologic conditions that greatly impact the lives of pediatric patients and their caregivers. The visible nature of these diseases negatively affects the self-image of children early in life as well as their relationships with their families and peers. Physicians recognize the importance of addressing both the physical and mental symptoms of their patients but are currently not equipped with clear guidelines to manage long-term psychosocial comorbidities in pediatric dermatologic patients. A PubMed and Google Scholar search of key words was conducted to explore self-image in pediatric patients with acne, AD, and psoriasis. Chronic skin diseases put pediatric patients at risk for strained family relationships, poor self-image, psychiatric comorbidities, stigmatization, and eventual suicidal behavior. A limitation of this study is a lack of a validated measure of quality of life in the pediatric population that fulfills enough criteria to evaluate long term quality of life in children and adults. Possible management options, including connecting patients with the same diagnosis and allocating resources to parents and teachers to better understand these chronic skin conditions, may provide pediatric patients with the support they need to develop resilience in the face of these challenges.

## 1. Introduction

Acne is one of the most frequent disorders seen by dermatologists and affects 80% of adolescents between the ages of 12–18 [1]. Among those who are genetically predisposed to acne vulgaris (AV), the pathogenesis involves both androgenic activity and specific ligands in the pilosebaceous unit that cause the proliferation of infrainfundibular sebocytes and keratinocytes and result in lipogenesis and comedogenesis [1]. Atopic dermatitis (AD) is a chronic inflammatory disorder that exists within the spectrum of atopic disease, including food and environmental allergies and asthma [2]. Eighty-five percent of AD cases start by age five and twenty-five percent of children have wheezing or eczema symptoms by their teen years [2]. Psoriasis is a chronic immune disease that involves T-cell mediated chronic inflammation of the skin [3]. Activated T cells and dendritic cells release cytokines, including TNF-alpha, IL-17, IL-23, and interferon gamma that cause a cascade of other cytokines, leading to the proliferation of keratinocytes [3]. The incidence of psoriasis in pediatric patients is unknown, but some 37% of adult patients with psoriasis develop symptoms before the age of 20 [3]. Chronic plaque psoriasis is the most common form of the condition, and males and females are equally affected [3].

All three of these conditions greatly impact the quality of life of patients and their caregivers. Some pediatric patients have disease that persists into adulthood, putting them at risk for life-long challenges, including increased stigmatization, psychiatric comorbidities, and suicide [2]. At the end of the last century, 16 cases of completed suicide among dermatology patients raised awareness about the potential psychological consequences of skin disease [4]. A minority of suicidal individuals seek help, so a consultation with a dermatologist might present an essential opportunity to identify suicidal ideation and to implement strategies to prevent suicidal behaviors [4]. There are several risk factors that put patients with skin conditions at a greater chance of suicide, including clinically significant emotional distress, poor body image, difficulties maintaining close relationships, and impaired quality of life [4]. Therefore, it is important to address impacts on quality of life secondary to disease severity as well as possible comorbid psychosocial conditions among pediatric patients with acne, AD, and psoriasis and their families to cement healthy coping mechanisms and positive self-perception for the future. The purpose of this systematic review is to discuss the impact of skin disease on quality of life (including on body image and self-esteem), increased stigmatization, and risk of eventual suicide in pediatric patients with acne, atopic dermatitis, and psoriasis.

## 2. Materials and Methods

A PubMed and Google Scholar search included the key words acne pathogenesis, psoriasis pathogenesis, atopic dermatitis pathogenesis, pediatric acne quality of life, pediatric psoriasis quality of life, pediatric atopic dermatitis quality of life, family relationships and atopic dermatitis, acne bullying in adolescents, stigmatization in children with atopic dermatitis, bullying in pediatric psoriasis patients, management of pediatric quality of life for acne, atopic dermatitis and psoriasis, suicide and acne in children, suicide in pediatric atopic dermatitis patients, and suicide in pediatric psoriasis patients. Articles were limited to studies on children ages 18 years and younger. Results included 13 articles on acne, 20 articles on AD, and 15 articles on psoriasis, which were prescreened by reviewing the abstracts. Additional articles were identified by reviewing reference lists in the key articles.

## 3. Results

In the following section, we will discuss the impact on quality of life, effect of bullying, and chance of suicidal behavior in pediatric patients with acne, AD, and psoriasis patients.

### 3.1. Acne

#### 3.1.1. Effect on Quality of Life

Pediatric patients with chronic dermatologic conditions involving visible lesions face challenges to their self-image as well as to the way others perceive them, which may negatively affect their daily life. Those with acquired skin conditions, such as AV, feel a more potent impact on self-esteem than those with congenital skin conditions [5]. In a study including adolescents aged 11 to 18 years, those with acquired dermatologic diseases affecting the face expressed a worse self-image than those with congenital facial diseases [6].

This negative self-perception among adolescents is not necessarily correlated with disease severity. Review studies on the psychosocial impact of AV acknowledge the discrepancy between the patient’s assessment of their acne severity and that performed by a medical professional [1]. The patient’s self-assessment was far more influenced by factors including anxiety, depression, and low self-esteem. Patients with the worst scores on self-esteem scales were those who believed they have more severe AV, despite incongruent medical evaluations [1].

However, other studies suggest a relationship between disease severity and quality of life. In a cross-sectional study of 23 high schools in Athens, Greece, the impact of acne on quality of life measured by the child daily life quality index (CDLQI) was proportional to acne severity (*p* < 0.0001) [7]. Differences in the CDLQI score were influenced by feelings of low self-esteem, disturbance from symptoms of acne, feelings of unworthiness in their relationships, and unpleasant treatment [7].

Poor self-esteem and feelings of unworthiness in childhood may put pediatric patients at greater risk of psychosocial comorbidities. In a study of 72 patients with mild-to-moderate acne on their face, the mean score for the Carroll Rating Scale for Depression (CRSD) was within the range of clinical depression [8]. Positive changes in self-perception may be achieved with successful medical intervention [9]. Fifty percent of acne patients who were treated with standard topical and systemic agents for three months reported fewer feelings of embarrassment, and 58% of these patients reported less social inhibition following treatment [9].

#### 3.1.2. Impact of Bullying

Pediatric patients do not have control over the way others treat them based on their appearance, which can pose additional challenges in their life and negatively impact their body image. In a study of pediatric patients aged 11 to 18 with either congenital facial abnormalities (*n* = 148) or acquired facial differences (*n* = 32), patients with acquired differences were more likely to report stigmatization (*p* < 0.0001) [5]. In a recent qualitative study of 62 semi-structured interviews, teasing, taunting, or bullying was a substantial problem for participants with acne, psoriasis, and atopic dermatitis [10]. Details mentioned in these interviews included the mean-spirited nature of the teasing; the use of teasing as a means of social exclusion, establishing or enforcing power relationships, as well as teasing related to the fear of infection; and the emotional consequences of this prolonged bullying [10]. Oftentimes the perpetrators of the teasing and bullying were other adolescents, except for a subsection of psoriasis patients who reported ridicule from health professionals [10]. Those who suffered teasing described the psychological consequences as primarily effects on their self-image and self-esteem [10].

Patients with acne may internalize negative messages from years of persistent bullying, and this may affect the way that they present themselves to others. In a recent survey study, 1002 adolescents and adults were asked to describe their first impressions when they were presented with images of teenagers with either clear skin or acne [11]. The image of the teenagers with clear skin received higher ratings on all positive characteristics (happy, healthy, intelligent, self-confident, fun, trustworthy, creative, popular, cool, athletic, and outspoken) and lower ratings on all negative characteristics (shy, nerdy, stressed, lonely, boring, unkempt, unhealthy, introverted, and rebellious) from both teenagers and adults than the images of teenagers with acne [11]. In response to the images of the teenagers with acne, both teenagers and adults believed that these teens were more likely to be shy, to be bullied, and would have more difficulty finding a job [11]. In the same study, when adolescents with acne were presented with a survey based on their self-perception, they reported less self-confidence, more difficulty finding romantic partners, more difficulty maintaining friendships, more challenges in school, and more difficulty getting a job [11].

#### 3.1.3. Risk of Suicide

Compared to the general population, depression, anxiety, and suicidal ideation are more common in patients with acne [9]. Adolescents are particularly at risk of these psychiatric disorders in part because of their susceptibility to criticism from peers [9]. The consequences of these disorders may be severe if they go undetected and untreated, as acne is an independent risk factor for suicide [9].

Patients with mild-to-moderate acne of the face experience a high prevalence of suicidal ideation (5.6%) and desires to be dead (8.3%) relative to the general population. The prevalence of suicidal ideation was found to be 8% in a Pakistani study of 50 patients ages 13–25 [12]. In New Zealand, 9567 students ages 12 to 18 years were surveyed to evaluate the relationship between acne, depressive symptoms, anxiety, and suicidal behaviors [13]. The survey statement “having really bad or terrible problem with acne” was found to be independently associated with severe depressive and anxiety symptoms, as well as with a higher risk of suicide attempts. The association between acne and suicide attempts remained after controlling for depressive and anxiety symptoms [13].

In a recent study of 3775 adolescents in Norway, nearly one in four of the participants with severe acne reported suicidal ideation [14]. The prevalence of suicidal ideation in those with severe acne was more than twice as high in girls and three times higher in boys compared to those with mild to moderate acne, suggesting the importance of early psychiatric and dermatologic intervention in cases of high disease severity [14]. This association between acne and suicidal ideation was controlled for depressive symptoms, family income, and ethnicity [14].

In the pursuit of symptomatic control in pediatric patients with acne, it is important for providers to be aware of the possible psychiatric adverse effects of certain acne treatments. Isotretinoin is a metabolite of vitamin A that is a common treatment for nodulocystic acne [15]. The effects of isotretinoin on the mature central nervous system are not well known. The psychiatric adverse effects of oral isotretinoin treatment have not been well studied in adolescents [15]. In a study of 60 adolescent patients with acne using isotretinoin, there was no effect on depression or suicidal ideation after three months of treatment, evidenced by no change in the Hospital Anxiety and Depression Scale (HADS) or the Suicide Probability Scale (SPS) scores [15]. There was an improvement in their quality of life, anxiety symptoms, and obsessive compulsive symptoms as evidenced by a decrease in their Acne Quality of Life Scale (AQLS), Liebowitz Social Anxiety Scale (LSAS), and Maudsley Obsessive Compulsive Question List (MOCQL) scores [15]. A possible limitation of this study is the short time frame within which the depressive and suicidal symptoms were monitored and a study population that may not be large enough to detect rare severe depression events.

### 3.2. Atopic Dermatitis

#### 3.2.1. Effect on Quality of Life

AD can affect children at much younger ages than acne can. Managing this chronic dermatologic condition puts notable strain on the caregivers of pediatric patients with AD, which often negatively impacts their relationship with their child [16]. Compared to controls, mothers of infants with AD more frequently characterized themselves as more depressed and anxious and described their infant more negatively than positively [17]. In a study evaluating family quality of life, most caretakers of pediatric patients with AD cited problems with exhaustion, frustration, guilt, helplessness, and resentment [18].

The depression and anxiety of the caretakers of pediatric patients with AD may cause these children to develop negative perceptions of themselves, which may impact their future quality of life [16]. Compared to peers without AD, preschool patients with AD have a greater incidence of behavioral issues, disrupted family dynamics, and anxious parenting [19]. Pediatric patients with AD exhibited similar behavioral problems to children with other severe chronic diseases such as renal failure, including increased dependency, fearfulness, and nighttime sleep disturbance, usually stemming from their chronic discomfort [19]. In a qualitative study of 55 pediatric patients with AD living in Hong Kong, most of the children listed their primary physical challenge as intense pruritus, which interfered with their sleep, diet, play, and sports. Frustrations with family members often revolved around parents telling the participants to stop scratching [20]. When the children heard these demands from their parents, they felt misunderstood and criticized as five of the participants reported being scolded and beaten for scratching [20]. This cycle of desperation and helplessness continued as the children resisted their parents’ demands to take their medications [20]. The children in this study understood the benefits of applying the ointments and taking the oral medications for their AD, but some of the treatments they were prescribed were uncomfortable for them to use [20].

The frequent disputes with their caretakers caused some children to put more pressure on themselves to perform better academically to meet parental expectations [20]. Other children internalized negative perceptions of their body over time, viewing themselves as “dirty” and “disgusting,” expressing hatred of their skin, or feeling the need to cover their skin to prevent others from seeing it [20]. In a cross-sectional study of 51 pediatric patients with AD and their guardians, the relationship between the severity of the child’s AD and the quality of life of the child and that of the caregivers was evaluated [21]. Greater severity of AD, measured by the Severity Scoring of Atopic Dermatitis (SCORAD) index, was associated with worse quality of life of the children and their caregivers, measured by the CDLQI and the Dermatitis Family Impact (DFI) scores (*p* < 0.001) [21]. Most of the children’s AD had a weak effect on the CDLQI scale, compared to their guardians, who fit into the moderate to very high effect on the DFI scale, which suggests a great level of compromise and strain among family members of pediatric patients with AD [21].

#### 3.2.2. Impact of Bullying

Once patients with AD begin to attend school, they often face teasing and bullying from their peers due to negative misconceptions or misinformation regarding their condition, and this stigmatization can have detrimental effects on their education, extracurricular activities, and future workplace productivity. Peers may avoid interacting with them due to beliefs that their rash is contagious, which may contribute to worsening self-esteem [5]. In a qualitative study of pediatric patients with AD in Hong Kong, participants cited responses from their classmates to their skin as a major source of daily stress (*n* = 17) [20]. Through semi-structured interviews and analysis of drawings, Chinese pediatric patients reported frequent stigmatization from their fellow classmates due to their skin condition [20]. Several students noted that they were verbally, socially, and physically bullied because of their AD. Potentially due to this early social exclusion, pediatric patients with severe AD are less likely to be involved in sports or outdoor activities [20]. Many of these patients with AD indicated that their teachers perpetuated this negative environment at school due to their limited knowledge of their skin disease, causing them to mistreat or completely ignore their students with AD [20].

Nearly 80% of pediatric patients with AD are clear of lesions by the onset of puberty [22]. For patients whose AD persists into their pre-teenage and teenage years, they may be presented with additional psychosocial challenges [16]. Older pediatric patients with AD report having fewer friends, participating in fewer social events and sports teams, and missing more class than their peers without AD [23]. Patients with AD who develop their condition later in adolescence are at a higher risk of having their disease persist into adulthood, potentially impacting their future work productivity and psychological wellbeing [22,24].

In a study using discreetly positioned cameras to analyze the personal space between people on a street in relation to those with visible skin conditions, personal space increased dramatically when someone with a visible skin disease was “planted” walking in the street [25]. Some of the subjects moved to the unaffected side of the planted person if they could not fully move away and others crossed the road to avoid the affected individual [25]. This study raised awareness about possible implicit biases towards those with visible dermatologic conditions and has important implications for health professionals who treat children with skin conditions to ensure that their own social conditioning does not affect the care of their patients [25].

#### 3.2.3. Risk of Suicide

Difficult family dynamics, increased stigmatization among peers, and negative self-image put pediatric patients with AD at risk for future psychosocial comorbidities and potentially suicidal behavior. Suicide is the tenth leading cause of death among Americans and the second leading cause of death among adolescents, and unfortunately the suicide rate is rising [26]. In a Korean study, pediatric patients with AD were at greater risk of suicidal ideation (odds ratio (OR), 1.23; 95% confidence interval (CI), 1.13–1.35) and suicide attempts (OR, 1.31; 95% CI, 1.12–1.52) [27]. In another study examining pediatric patients with AD compared to controls without AD, female pediatric patients with AD had an increased risk of suicidal ideation (OR, 1.114; 95% CI, 1.046–1.186) and suicide attempts (adjusted OR, 1.188; 95% CI, 1.065–1.325) [28]. In a study of 788,411 adolescents in South Korea, they reported an association between AD and suicidal ideation and attempts [28]. Among the 22% of adolescents with AD, 34.7% reported depression, 19% reported suicide ideation, and 4.5% reported suicide attempts. The most strongly influencing factors for suicidal ideation and depression were perceived stress and unhappiness related to their dermatologic condition [28].

In a study evaluating the relationship between AD and suicidal behaviors, 74,186 South Korean adolescents in middle and high school completed the Eighth Korea Youth Risk Behavior Web-Based Survey and demonstrated an association between AD and suicidality, particularly among females with a distorted perception of their weight [29]. Females with AD were more likely than males to report suicidal behaviors, depressed mood, and increased stress, as well as to overestimate their body weight (*p* < 0.001) [29]. Distorted weight perception, either underestimation or overestimation, was associated with suicidal ideation, planning, and attempts among males and females [29]. Female adolescents with AD were more likely to report suicidal ideation, planning, and attempts (*p* <  0.001), with females with an overestimated weight perception exhibiting the highest risk [29]. Male adolescents with AD were only more likely to report suicidal ideation [29].

In the study of 74,186 Korean adolescents, results were adjusted for other psychological comorbidities related to suicide, implicating an alternative, possibly physiologic mechanism mediating the association between AD and suicidal behaviors [29]. AD is associated with higher levels of proinflammatory cytokines, which may contribute to the association with suicidality in patients with AD by altering the metabolism of serotonin as well as the balance of neurotransmitters in the brain [30]. Higher levels of proinflammatory cytokines were measured in the cerebrospinal fluid of patients who attempted suicide, suggesting a possible relationship between inflammatory cytokines and suicidality (*n* = 47) [31]. Additionally, in patients with AD receiving treatments targeting proinflammatory cytokines, there was a decrease in depressive and anxiety symptoms; however, this effect has not yet been demonstrated in the pediatric population. Moreover, the associations do not necessarily imply causation [32].

The effects of anxiety and depression may be a possible explanation for the increased prevalence of suicidality among patients with AD; however, there is some speculation that the systemic type 2 immune response in AD may play a role in altering the maturation of the pediatric brain [33]. Proinflammatory cytokines inhibit elongase enzymes and shorten lipid species involved in skin barrier function [33]. It is still unknown whether the systemic type 2 inflammation influences fatty acid elongation and composition in the brain, especially during early infancy when the blood–brain barrier demonstrates increased permeability [33]. Proinflammatory cytokines, such as interleukin (IL)-4 and IL-13, have not been shown to cross the blood–brain barrier in mature animals, but they may be produced in microglia [33]. Several laboratory studies reported a reduced adrenocortical stress response in pediatric patients with AD compared with healthy controls, possibly impeding these children’s ability to adapt to stressful situations and increasing their risk of suicide [34]. Proinflammatory cytokines may also worsen depressive moods in patients with AD by activating the hypothalamic–pituitary–adrenal (HPA) axis, chronically increasing corticotropin-releasing hormone and cortisol, which detrimentally affect neurons [32].

### 3.3. Psoriasis

#### 3.3.1. Impact on Quality of Life

Among children aged 5–16, psoriasis decreased health-related quality of life (HRQL) by 30.5% (*p* < 0.001) [35]. Psoriasis affected HRQL more than epilepsy, enuresis, and diabetes. In a similar study of 118 children aged 5–16 years surveyed with the CDLQI, psoriasis and atopic dermatitis had a greater impact on HRQL compared to other chronic dermatologic conditions, such as vitiligo [35]. In a qualitative study of 32 adolescent dermatology patients ages 12–19 years, those with psoriasis were affected by the greatest range of different HRQL domains, including psychological, social, and physical [36]. The psychological domain specifically focused on emotional aspects, bullying, self-esteem, and judgement from others [36]. Children with psoriasis reported the greatest impairment of quality of life based on scoring from the CDLQI, followed by those with atopic dermatitis [3]. The CDLQI scores for pediatric psoriasis patients ranked higher than those for alopecia, localized eczema, acne, and urticaria patients [3].

Psoriasis may impact the lives of both the patients and their caregivers. In a multicenter study of 129 pediatric patients with psoriasis and their caregivers, the CDLQI and DFI were used to evaluate the effect of psoriasis on the quality of life of the children and their family members [37]. The average CDLQI score was 7.6, suggesting a moderate effect on the quality of life of the patients, with personal feelings being the most severely impacted domain [37]. Emotions were the most severely impaired domain for caregivers based on the DFI. The DFI score was positively correlated with the CDLQI score (r = 0.554, *p* < 0.001) and the PASI (r = 0.350, *p* < 0.001) [37]. Among patients with more rigorous treatment regimens, including systemic agents and phototherapy, there was impairment in multiple domains for the quality of life of their caregivers compared to those receiving first-line therapies [37]. Caretakers of pediatric patients with psoriasis experience psychological strain due to the unpredictable pattern of their child’s disease, leading to feelings of helplessness [38]. They often report frustration over dealing with their child’s low mood and dissatisfaction over medical care [38]. Some caregivers felt overwhelmed by the responsibility of caring for their child’s condition and described compromising their own personal health for the sake of their family [38].

Adolescent patients with psoriasis frequently face challenges regarding poor self-esteem, difficulties with sexual intimacy, stigmatization, and strained family and social relationships [35]. Psoriasis especially impacts the types of physical activities adolescents engage in, particularly extracurricular sports, which may negatively contribute to other comorbidities associated with psoriasis, including diabetes, obesity, hypertension, and psychiatric disorders [35]. The visible nature of this skin condition and its associated discomfort both contribute to the avoidance of physical activity in this age group [35]. In an online focus group, 48% of adolescent participants cited the visibility as the “worst” aspect of psoriasis as well as the loss of control since psoriatic lesions are often recurrent and unpredictable [35]. These negative thoughts about their bodies may affect the self-esteem of adolescents over time and put them at a higher risk of psychiatric conditions such as depression and anxiety. Compared to healthy controls, self-esteem (SE) (*p* < 0.001) and body image (BI) (*p* = 0.021) were lower in patients with psoriasis [39]. Psoriasis Area and Severity Index (PASI) was negatively correlated with BI (r = −0.423) but positively correlated with the quality of life (*r* = 0.703) and SE (*r* = 0.448). Another study demonstrated that poor SE in patients with psoriasis was highly associated with comorbid psychopathologies, such as sexual dysfunction disorders, anxiety, depression, and suicidality. Educational status did not affect DLQI scores in psoriasis patients; however, SE improved with higher education levels (*p* < 0.05) [39].

Psoriasis especially affects appearance, confidence, and lifestyle in adolescent patients. Adolescent patients report feelings of loss of control because of the unpredictable course their disease, which impedes their ability to cope [3]. They agree that self-esteem issues stemming from psoriasis stay with them in the long term, sometimes after complete clearance [3]. Some of the most common coping mechanisms are avoidance behaviors or concealing strategies, which may affect their daily activities or intimacy with sexual partners. Talking with peers helps some adolescents with their confidence, feelings of loneliness, and their knowledge of other coping mechanisms [3]. The main contributors to depression among patients with psoriasis were female gender, poor perception of their appearance, poor self-worth, increased psychological distress, and reduced emotional social support [40]. Therefore, achieving better/complete clearance through proper treatment may positively affect their body image and may prevent the development of other psychiatric conditions such as depression [40].

#### 3.3.2. Impact of Bullying

Certain qualities, including age and gender, may affect the level of stigmatization among patients with psoriasis. In a qualitative study of patients with psoriasis, older respondents heard fewer harsh comments about their condition and experienced less frequent thoughts about their disease being contagious, namely thoughts that others were uncomfortable touching their skin lesions, or that others avoided them due to their illness [41]. These results are consistent with the higher degree of stigmatization in younger persons with psoriasis and atopic dermatitis compared to older patients with the same conditions [42]. Females felt a stronger sense of stigmatization regarding their psoriatic lesions compared to men [41].

Internalized stigma occurs when the individual accepts negative stereotypes about the illness created by society and withdraws himself/herself, leading to feelings of worthlessness, decreased self-esteem, poor quality of life, increased depression, and suicidality [43]. In a multicenter study comparing 125 pediatric and 1235 adult patients with psoriasis, higher levels of internalized stigma were found in the pediatric group, associated with poor quality of life, general health status, and psychological disorders [43]. Internalized stigma contributes to psychosocial impairment, which may negatively affect the utilization of health care and treatment.

#### 3.3.3. Risk of Suicide

Poor quality of life, negative self-perception, and increased stigma all put patients with psoriasis at risk for psychiatric comorbidities and possible suicidal behavior. In a single-center observational study, 217 patients with psoriasis were screened for depression and suicidal ideation to investigate the association between psoriasis and psychiatric comorbidities [44]. Patients with severe psoriasis had higher scores on the CRSD than did patients with less-severe psoriasis (*p* < 0.001) [44]. About 7% of patients with severe psoriasis had active suicidal ideations compared to 4% in the general population [44]. A population-based study with data from the National Health and Nutrition Examination Survey (NHANES) found an association between psoriasis and depression even after controlling for sex, age, race, body mass index, physical activity, history of smoking, alcohol use, myocardial infarction, stroke, and diabetes mellitus (*p* < 0.001) [44].

In a cross-sectional, multicenter study of dermatology patients, a greater number of patients with psoriasis (13.8%) demonstrated clinical depression, as determined by the HADS, compared to healthy controls (4.3%) (adjusted OR, 3.02; 95% CI, 1.86–4.90) [44]. Episodes of self-injurious behavior (SIB) were higher in psoriasis patients compared with healthy controls (adjusted OR, 1.94; 95% CI, 1.33–2.82) [45]. Among patients with psoriasis expressing SIB, 67.6% indicated that their suicidal thoughts were due to their skin disease [44]. In a cohort of patients with psoriasis in the United Kingdom, major depression was identified as a risk factor that increased the rate of SIB by 10-fold among patients with psoriasis compared to the general population [44]. Other risk factors for suicide in this population include younger age, increased severity, and concomitant psoriatic arthritis [45].

Although females experience greater distress over the appearance of psoriatic lesions than males, both genders have an equal risk of depression and suicidality [45]. In a population-based cohort study in Denmark, there was an increased risk of suicide attempts in patients with severe psoriasis compared to those with less severe disease (*p* = 0.049) [44]. In a retrospective cohort study in the United Kingdom, there was an increase in completed suicides in patients with psoriasis compared to control patients (relative risk, 1.3; 95% CI, 1.0–1.8) [44].

Biological changes and poor self-image both contribute to the increased risk of suicide in this population. Proinflammatory cytokines involved in psoriasis may worsen depression [45]. Psoriasis leads to an immune-mediated increase in multiple proinflammatory cytokines, including IL-1 and IL-6 [46]. These cytokines over-activate the HPA axis and disrupt the negative feedback inhibition from corticosteroids including cortisol. This loss of inhibition results in decreased serotonergic neurotransmitter levels, which contribute to major depressive disorder [46]. Patients with psoriasis receiving interferon therapy for hepatitis C may experience flares of their skin condition along with depression as a side effect [46]. Patients with psoriasis treated with tumor necrosis factor (TNF) inhibitors or IL-12 and -23 inhibitors report improved mood [45]. Aside from biological changes, the appearance and discomfort of psoriatic lesions negatively impact both self-image and quality of life [45].

## 4. Discussion

Acne, AD, and psoriasis are chronic dermatologic conditions that may negatively impact the lives of pediatric patients and their caregivers. Patients with these conditions are forced to cope with visible lesions or chronic discomfort, which may negatively affect their self-image, activities of daily living, and interactions with their peers. In order to mitigate these negative feelings or discomfort, pediatric patients may avoid going to school, choose to wear different clothes, or participate in different extracurricular activities [20,22,23,24]. These patients may suffer from poor self-esteem, which increases their risk of being bullied by their peers at school as well as their risk of comorbid psychosocial conditions [1,2,3,4,5,6,7,8,9,10,11,12,13,14,15,16,17,18,19,20,21,22,23,24,25,26,27,28,35,36,37,38,39,40,41,42]. Those who are unable to develop healthy coping skills or who do not seek help for their psychosocial conditions face an increased risk of suicidal behavior [12,13,14,15,26,27,28,29,30,44,46].

A strength of this study is the large sample size of pediatric patients, especially in studies assessing the risk of suicide, as well as the mix of quantitative and qualitative data available. A limitation of this study is the lack of inclusion of other mental disorders with dermatologic symptoms that contribute to psychosocial sequalae in the pediatric population, including pathologic skin picking (PSP). PSP is associated with younger aged patients, increased risk of psychiatric symptoms, and worse psychosocial outcomes [47] The severity of dermatologic symptoms correlates with that of psychiatric symptoms in these patients [47]. In a recent study of 60 subjects with PSP, current comorbid Axis I psychiatric conditions were found in 38.3% of patients [48]. These psychiatric conditions included trichotillomania, compulsive nail biting, depressive disorder, and obsessive compulsive disorder [48]. Unfortunately, few of these patients ever sought treatment for their psychiatric conditions [48]. The following section describes several management options that may allow for early intervention in pediatric patients with acne, AD, or psoriasis to mitigate potential negative psychosocial sequalae.

In order to address the possible psychological ramifications of these skin diseases, it may benefit health care providers and patients to manage the physical and mental symptoms of their patient [9]. Some providers may underestimate the level of psychological distress of their patients, or not have access to adequate resources to manage these comorbid psychological conditions [9]. Nearly 84% of patients with AD and their caregivers did not know support groups for these skin conditions existed, and 74% reported that their provider had never discussed the psychological impact of their disease [9]. Over 50% of patients with AD are not discussing their quality of life with their physician and report feeling unsupported by their provider, suggesting the importance of communication surrounding the psychological impact of their skin disease on their daily life [9]. Administering a quality-of-life scoring system as an extra measure throughout clinical management allows opportunities for early recognition to address issues pertaining to conflict resolution, coping mechanisms, and quality of life that may impede better medication adherence among patients or caregivers [21].

### 4.1. Management Options

Multifactorial treatment of children with chronic skin diseases may help to prevent psychosocial sequalae [20]. It is important to treat the physical symptoms to reduce the discomfort of the patient and the possible stigmatization they receive from their visible lesions. Screening procedures to identify psychological issues in these children will also allow early intervention with mental health resources that assist with coping skills and resilience to help these patients maintain a positive self-perception in the face of adversity [20].

The Society for Adolescent Medicine’s bullying and peer victimization position paper encourages health care providers to take action when they suspect bullying or victimization behaviors, but there is no direct evidence to guide dermatologists on how to proceed [45]. Appropriate management of the physical symptoms may reduce bullying, given that teasing is often appearance-related, but the data are conflicting on whether improvement in disease severity correlates with reduced bullying [45]. Some schools have implemented peer-support groups as a means to confront bullying and its sequelae, but evidence on its efficacy is inconsistent [45]. However, in a randomized controlled trial, patients with AD and parent support groups and educational programs significantly improved scores on the “personal relationships” section of the CDLQI of patients with AD, suggesting the possible benefit of these interventions [45].

#### 4.1.1. Resources for Families

Connecting families of patients with AD with resources to educate them on their child’s condition and to develop healthy coping skills and conflict resolution may help create a more peaceful home [20]. Intervention services may be provided in a parallel format to both the patient and their caregivers with content that integrates AD-related educational components and psychosocial components [20].

#### 4.1.2. Resources for Teachers

Educating teachers about conditions such as AD as well as ways to deal with bullying in their classroom may be effective in developing a supportive school environment [20]. Teachers’ mistreatment of students with visible skin conditions negatively impacts the psychological wellbeing of these students and encourages other classmates to imitate their behavior [20]. School-based programs directed towards training teachers about chronic dermatologic conditions, recognizing the vulnerabilities of children with chronic dermatologic conditions, and encouraging them to teach their own students about these diseases may mitigate some of these potential harms [20]. The Tools for School Kit created by the National Education Association (NEA) was designed for teachers, parents, and students to learn more about AD and a similar kit for pediatric patients with psoriasis was created by the National Psoriasis Foundation [45].

#### 4.1.3. Physician-Mediated Interventions

Physicians can also help patients by connecting them with other children with the same disease to help them feel less isolated [35]. A number of condition-specific, in-person or online support groups exist that can help children connect with peers. Online forums may be especially helpful since they eliminate the stress of appearance, and the anonymity helps them express their feelings freely [35]. This intervention was successful in adolescents with HIV [35]. Dermatologists may also refer patients to specialized summer camps for children with skin disorders, such as Camp Discovery sponsored by the American Academy of Dermatology [46]. A multidisciplinary training program with pediatric and adolescent patients with psoriasis consisting of four sessions led by dermatologists or psychologists was recently developed to strengthen resiliency and coping skills among patients with psoriasis in an outpatient setting [49].

Shared decision-making (SDM), or an approach where physicians and patients make decisions together based on the best available data, is a possible method to involve patients in their own care, allowing the provider to address the therapeutic goals specific to their patient and to develop a strong patient–physician relationship [9,50]. This form of management provides the patient and their caregiver with additional education on the available treatment options and allows the patient to choose the treatment method that aligns with their priorities [9]. The education central to SDM along with its increased patient interaction and perception of customization may lead to increased medication adherence, which could reduce disease severity and improve psychological conditions [9,50,51].

There are a number of scoring systems to evaluate quality of life in both patients with chronic dermatologic conditions and their caregivers, including the CDLQI, Infants’ Dermatitis Quality of Life Index (IDQoL) DFI, and the Childhood Atopic Dermatitis Impact Scale (CADIS); however, none of these measurement tools fulfill enough criteria to evaluate the long-term quality of life in children and caregivers and can be recommended for use (Table 1) [9]. The Global Research on the Impact of Dermatological Diseases (GRIDD) project is currently developing a patient-impact measurement tool (PRIDD) with the goal to address the issues with current QoL measurement tools, which will allow for early psychiatric intervention among pediatric patients and their families and hopefully mitigate future psychosocial consequences [9].

## 5. Conclusions

Acne, AD, and psoriasis are common dermatologic conditions that affect the pediatric population and have a great impact on the quality of life of patients and their caregivers. Patients face increased risk of poor self-esteem, stigmatization, and psychosocial conditions, which influence their daily activities at home, school, and work [1,2,3,4,5,6,7,17,18,19,20,21,22,23,24,25,35,36,37,38,39,40,41,43]. Patients develop a number of coping skills to mitigate these negative experiences including avoidance and wearing more conservative clothing, which may affect their attendance at school as well as their choice of extracurricular activities. [20,22,23,24]. Those who are unable to develop healthy coping skills are at a greater risk of worsening psychosocial conditions, including depression and anxiety, which may increase their risk of suicidal behavior [12,13,14,15,26,27,28,29,30,44,46].

Although physicians recognize the importance of addressing both the physical and the mental symptoms of their patients, there are currently no clear guidelines about how to manage long-term psychosocial comorbidities in pediatric patients with dermatologic conditions. Some possible physician-mediated interventions include connecting patients with the same disease, informing their patients and caregivers about support groups, multidisciplinary training programs, and SDM [9,20,35]. Other possible interventions are those directed towards helping families to develop healthier coping skills and conflict resolution, as well as training programs for teachers to better understand the symptoms of children with chronic dermatologic conditions and how to reduce bullying behaviors in their classrooms [20]. Multifaceted treatment focused on controlling physical symptoms along with interventions to build resilience and positive self-perception may equip pediatric patients with dermatologic conditions with the means to maintain a good quality of life and face future challenges.

## Figures and Tables

**Table 1:** Current Quality of Life Measurement Tools for Pediatric Dermatologic Patients.

Name	Participants	Description	Scoring Algorithm	Severity Scoring	Advantages	Limitations
Children’s Dermatology Life Quality Index	Children aged 4–16 [52]	10 questions in a 1-week recall period [53] Includes questions about symptoms, embarrassment, friendships, clothes, playing, sports, school, bullying, and impact of treatment [52]	0–3 for each question 0: Not at all 1: Only a little 2: Quite a lot 3: Very Much [52]	0–1: no effect 2–6: small effect 7–12: moderate effect 13–18: Very large effect 19–30: extremely large effect [52]	Measures QoL in children with other comorbid nonskin diseases Allows comparison between children with many different skin diseases [52]	Does not correlate well with acute and chronic AD severity scores, including SCORAD and total CDLQI [53]
Infant Dermatitis Quality of Life Index	Infants with AD less than 4 years of age [54]	10 questions in a 1-week recall period [55] Includes questions about itching, mood, sleep, play, family, activities, mealtime, dressing, bathing, and problems from treatment of disease [54] Question for parents to assess disease severity [54]	Q1/5–10: 0: None 1: A little 2: A lot 3: All the time [55] Q2: 0: Happy 1: Slightly fretful 2: Very fretful 3: Always crying Q3: 0: 1–15 min 1: 15 min–1 h 2: 1–2 h 3: >2 h Q4: 0: < 1 h 1: 1–2 h 2: 3–4 h 3: >5 h	0–30 The higher the score, the greater the impact on QoL Severity scored separately 4: Extremely severe 3: Severe 2: Average 1: Fairly good 0: None [55]	Easy and quick to administer in an outpatient setting 50 parents of infants with AD completed IDQoLIs before the 1st and 2nd dermatology consultations and demonstrated a decrease in median IDqoLI score from 8 to 5.5 [56]	Only assesses effects on QoL in patients with AD Only used as a short-term measurement
Dermatitis Family Impact	Caretakers of pediatric patients with AD [54]	10 questions in a 1-week recall period Includes questions about housework, feeding, sleep, family activities, time spent on shopping, expenses related to treatment, fatigue, emotional distress, and family relationships [52]	0–3 for each question. 0: Not at all [52] 1: Only a little 2: Quite a lot 3: Very Much	0–30 The higher the score, the greater the impact on QoL [52]	Easy and quick to administer in an outpatient setting 50 parents of infants with AD completed DFIs before the 1st and 2nd dermatology consultations and demonstrated a decrease in the median DFI score from 9 to 3 [54]	Only assesses effects on QoL among family members of patients with AD Only used as a short-term measurement
Childhood Atopic Dermatitis Impact Scale	Children with AD younger than 6 years of age and their parents [57]	45-item questionnaire in a 4-week recall period Includes 5 domains: child symptoms, child activity limitations and behavior, family and social function, parent sleep, and parent emotions [57]	0–4 for each question Related to frequency from never to all the time [57]	Score of 0–180 The higher the score, the greater the impact on QoL [58]	Positive test–retest reliability, concurrent validity, discriminative validity, responsiveness evaluation Assesses QoL of both patients and caretakers [57]	Only pertains to patients and caregivers of those with AD Lack of a gold standard to use in comparison with global health questions [58]

AD, atopic dermatitis; CDLQI, Children’s Dermatology Life Quality Index; IDQoLI, infant dermatitis quality of life index; QoL, quality of life; Q1/5–10, questions 1 and 5–10; Q2, question 2; Q3, question 3; h, hours; min, minutes; Q4, question 4; SCORAD, scoring atopic dermatitis.
